# Lower limb varicose veins in free fibula flap reconstruction: a surgical case report and scoping review

**DOI:** 10.1080/23320885.2026.2742738

**Published:** 2026-10-07

**Authors:** Sebastian Holm, Reza Tabrisi

**Affiliations:** a Department of Reconstructive Plastic Surgery, Karolinska University Hospital, Stockholm, Sweden; b Faculty of Medicine and Health, Örebro University, Sweden; c Migraine Surgery Centre Scandinavia, Gothenburg, Sweden

**Keywords:** Free fibula flap, salvage, varicose veins

## Abstract

**Background:**

Mandibular reconstruction using a vascularized free fibular flap (FFF) remains a gold standard following oral cancer resection. Lower-limb varicose veins are a common manifestation of chronic venous disease, but their potential impact on free-flap viability is largely unexplored in the surgical literature.

**Case presentation:**

We present a case of FFF reconstruction in a patient with lower extremity varicose veins, detailing the intraoperative management of venous congestion and the postoperative course.

**Methods:**

We conducted a scoping review to identify publications addressing venous disease or abnormal venous findings in the context of FFF reconstruction.

**Results:**

The scoping review identified only two relevant publications.

**Conclusion:**

This report identifies an evidence gap regarding the relevance of donor-site venous findings in FFF reconstruction. The present case and limited literature are hypothesis-generating and do not establish a causal relationship between superficial varicose veins and flap compromise. Further research is needed to clarify whether targeted venous assessment has a role in selected patients.

## Introduction

Cancer of the oral cavity ranks as the sixth most common malignancy worldwide [1]. For extensive head-and-neck defects, free flap reconstruction remains the gold standard, with the free fibular flap (FFF), a vascularized fibular bone segment, with or without a skin paddle, serving as a primary option for major bone reconstruction [2]. With a reported 95% graft survival rate in the literature, preoperative vascular assessment of the lower extremities is critical to ensure a successful FFF harvest [3,4].

Current preoperative donor-site assessment for FFF reconstruction commonly focuses on arterial anatomy and limb perfusion using computed tomography angiography (CTA), magnetic resonance angiography (MRI), or duplex ultrasonography [5]. In contrast, the clinical relevance of pre-existing lower limb venous disease for FFF harvest and flap drainage is incompletely understood. Varicose veins are acquired dilated superficial veins, usually associated with chronic venous disease; they should not be equated with congenital vascular malformations, which are developmental abnormalities of vascular formation. Furthermore, visible superficial varices do not by themselves establish pathology of the deep peroneal venous system. Varicose veins should be distinguished conceptually from chronic venous insufficiency, which represents a broader spectrum of chronic venous disease and has been associated with cardiovascular comorbidity and mortality [6].

Measures used for venous thromboembolism prophylaxis, such as low molecular weight heparin (LMWH), should be distinguished from interventions intended to salvage an established free flap venous outflow problem [7–10]. Salvage of venous congestion may involve prompt exploration of the anastomosis, thrombectomy or thrombolysis in selected settings, revision or addition of venous anastomoses, venous supercharging, relief of mechanical compression, or controlled external bloodletting techniques when appropriate [11–18]. Pharmacological thromboprophylaxis may be part of perioperative care but does not correct chronic venous disease or replace mechanical restoration of flap venous drainage.

We present a surgical case report of mandibular reconstruction with an osteocutaneous FFF in a patient with clinically apparent lower limb varicose veins, followed by a scoping review of published reports addressing venous disease or abnormal venous anatomy in the context of FFF reconstruction. The aims were to: (i) map the available evidence regarding reported venous abnormalities relevant to FFF harvest or drainage; (ii) describe the intraoperative management and clinical course of the present case; and (iii) identify evidence gaps regarding donor site venous assessment and management. This case report has been reported in line with the SCARE 2023 guideline.

## Case description

### Background

A 64-year-old male patient with squamous cell carcinoma of the oral cavity (pT3N0) presented to our hospital for tumour resection followed by mandibular reconstruction. His medical history was notable only for a 42-year smoking habit and bilateral lower extremity varicose veins.

### History and indication for surgery

Recurrent oral verrucous lesions had been monitored and biopsied between 2011 and 2020. In 2020, a biopsy resulted in infection and subsequent fistula formation; even after wound healing, the patient reported persistent pain for 18 months with no clear origin (Figure 1C). In 2021, a mandibular biopsy revealed a well-differentiated squamous cell carcinoma (pT3N0). MRI and CTA (Figures 1A and 1B) showed lesions on the floor of the mouth involving both right and left mandible, indicating a trans-midline bilateral mandibular malignancy. Following multidisciplinary discussion, the patient was admitted for tumour resection and mandibular reconstruction.

**Figure 1.**
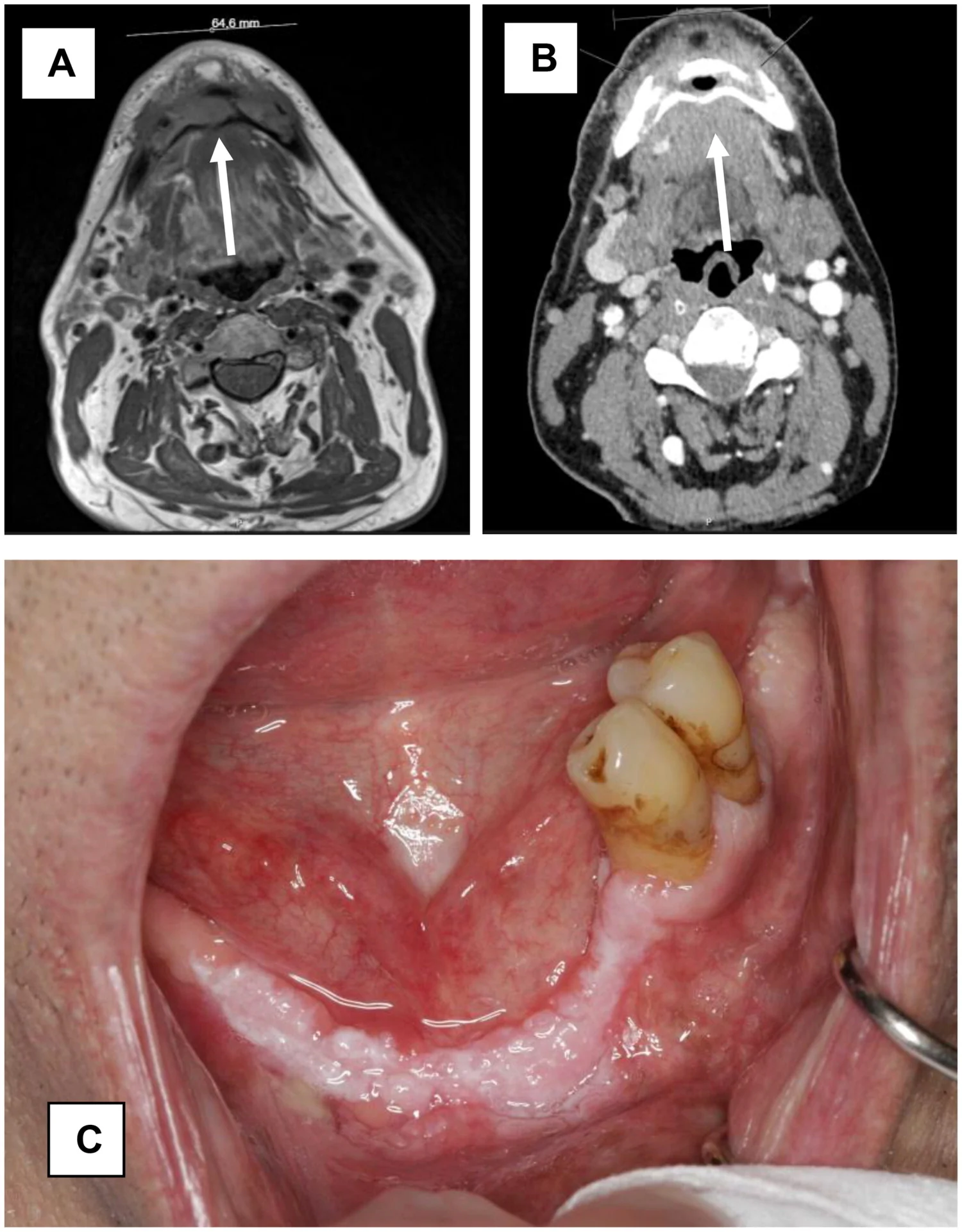
(A) Magnetic resonance imaging and (B) computed tomography angiography demonstrating the floor-of-mouth tumour with trans-midline mandibular involvement (white arrows). (C) Preoperative intraoral photograph demonstrating the lesion involving the floor of the mouth and bilateral mandibular region.

### Preoperative assessment

CTA confirmed adequate arterial flow in both lower extremities (Figure 2C), although Doppler signals were suboptimal. The left fibula was selected as the donor site because the patient had previously undergone multiple varicose-vein procedures on the right lower limb. Clinically visible superficial varicose veins were present on the left leg. These superficial varicose veins were also visible on the preoperative CTA (Figures 2A and 2B). Preoperative CTA was performed primarily for arterial assessment and did not include a dedicated venous duplex protocol. Therefore, reflux, patency, valve competence, and calibre of the deep peroneal veins were not systematically documented preoperatively. The intraoperative venous drainage problem could not be attributed with certainty to the superficial varicose disease.

**Figure 2.**
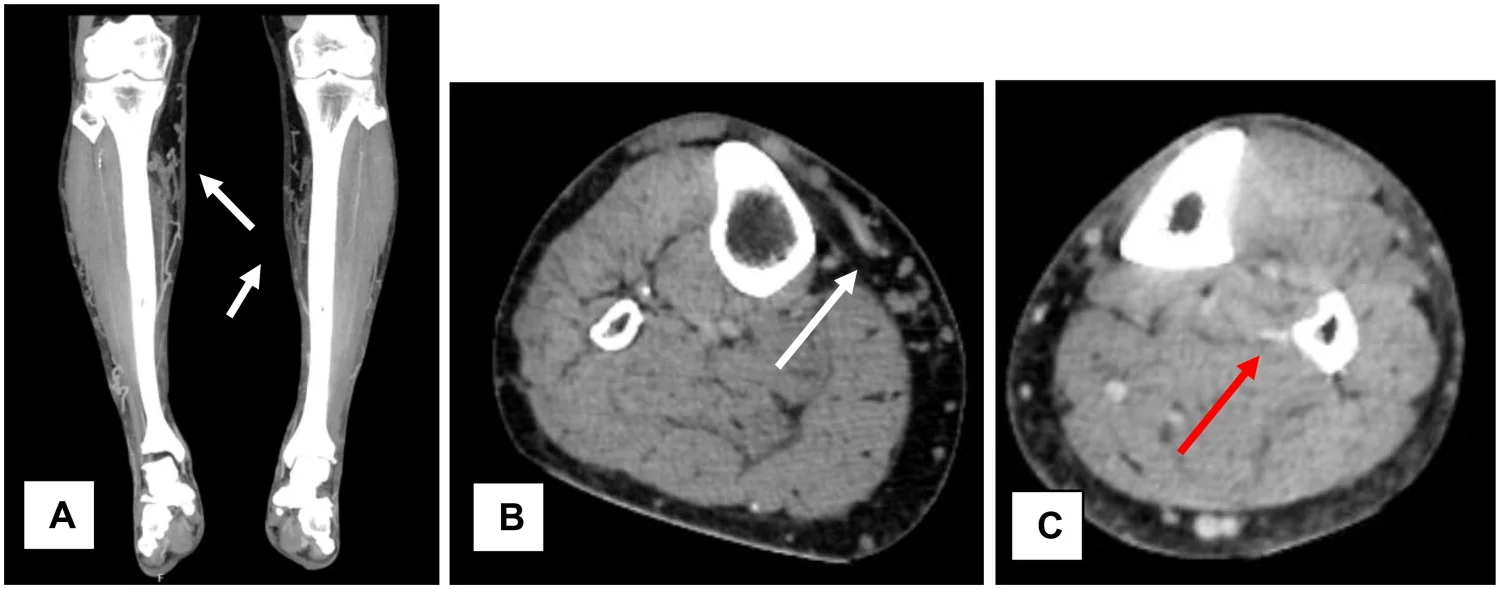
(A) Computed tomography angiography of the lower limbs demonstrating superficial varicose veins (white arrows). (B) Superficial varicose veins of the right lower limb (white arrow). (C) Preoperative computed tomography angiography of the left lower limb demonstrating the peroneal artery (red arrow).

### Flap harvest

The procedure was performed by an experienced microsurgeon. An osteocutaneous flap was chosen to cover the trans-midline bilateral mandibular defect. The left fibula, approximately 25 cm long with a 15 × 5 cm skin paddle (Figure 3), was dissected over 123 min under a 350 mmHg tourniquet. After flap elevation, the tourniquet was released and the flap remained perfused through its native pedicle for approximately 3 h before pedicle division.

**Figure 3.**
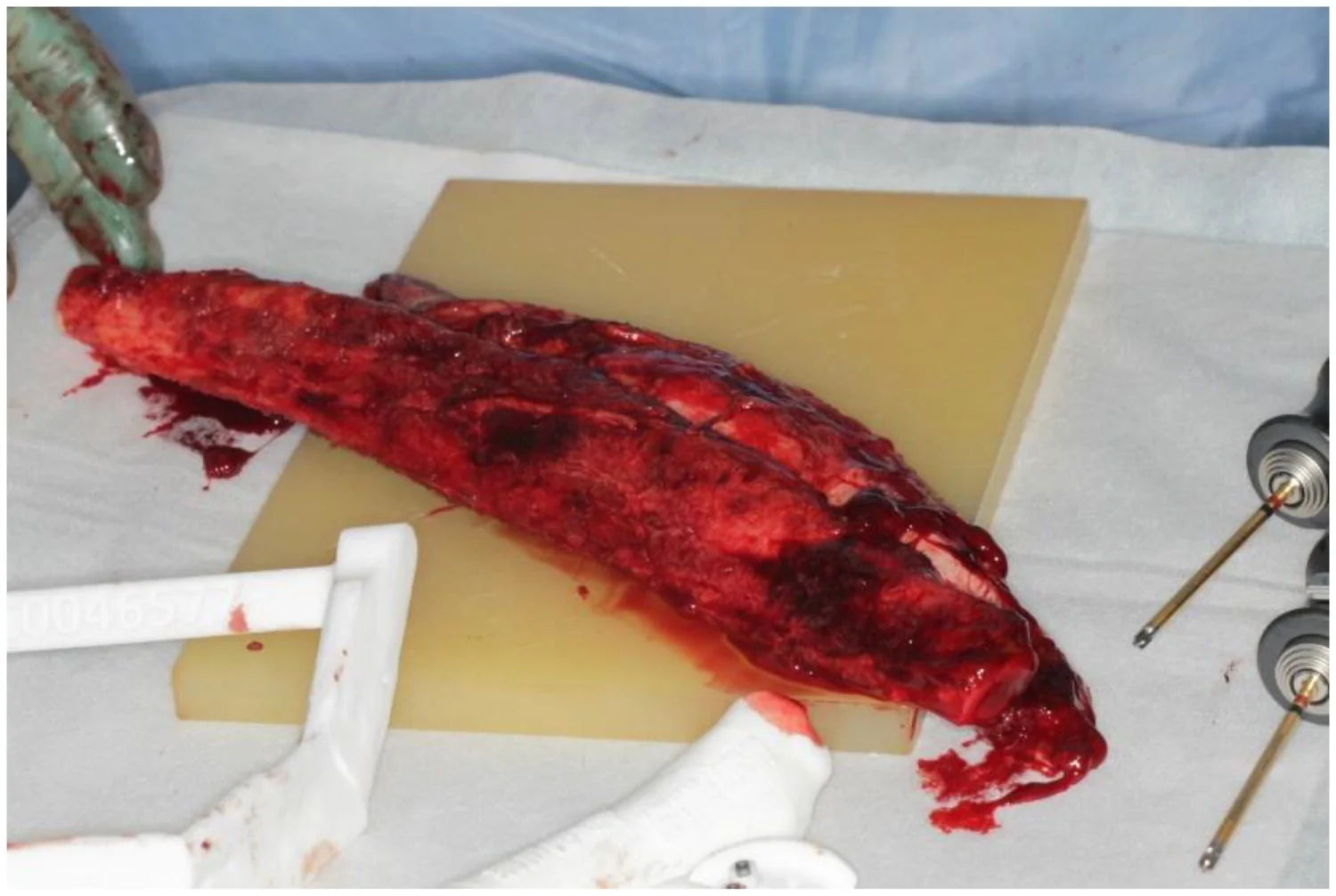
Harvested left fibula, ∼25 cm in length with a 15 × 5 cm skin paddle.

The peroneal artery was anastomosed end-to-end to the facial artery using 8-0 nylon sutures (Ethilon^™^, Ethicon, Johnson & Johnson MedTech, Somerville, NJ, USA). Two peroneal veins were anastomosed to the facial vein and external jugular vein using microvascular anastomotic couplers (GEM^™^ Coupler System, Synovis Micro Companies Alliance, Birmingham, AL, USA), measuring 3.0 and 3.5 mm, respectively. Total ischemia time was 275 min.

Intraoperative ICG fluorescence angiography (Verdye 5 mg/ml) demonstrated delayed venous washout from the skin paddle, and dark blood expressed from the paddle edges was consistent with venous congestion. The anastomosis between the first peroneal vein and the external jugular vein, initially constructed with a 3.0 mm coupler, was explored and revised. No thrombus or technical defect of the coupler was identified. The recipient and donor vein diameters were reassessed intraoperatively, and a 2.0 mm coupler was selected because the estimated effective vessel diameter was smaller than initially anticipated. The vein was irrigated with heparinized saline (50 IU/mL), and the anastomosis was reconstructed. The revision added 20 min of ischemia time.

Following revision, venous drainage improved clinically and on repeat ICG assessment; however, the relative contribution of coupler size, correction of vessel geometry, removal of a mechanical kink, or other factors could not be determined.

Although venous drainage improved after anastomotic revision, perfusion of the skin paddle remained clinically sluggish. The skin paddle was de-epithelialized and multiple drainage holes were created using an 18 G (1.3 mm) needle. Repeat fluorescence demonstrated improved perfusion, and Doppler confirmed arterial flow. The drainage holes were placed superficially in selected areas of the de-epithelialized paddle under direct visualization, avoiding visible perforators and the known course of intraflap vessels. Haemostasis was checked repeatedly, and the flap was monitored closely for bleeding or haematoma formation.

### Postoperative course

Venous thromboembolism prophylaxis consisted of dalteparin 5000 IU, administered subcutaneously once daily, beginning 4 h after surgery and continued for 10 days. This regimen was prescribed as postoperative venous thromboembolism prophylaxis and not as treatment of chronic venous disease or established flap thrombosis.

Acetylsalicylic acid was administered as a 300 mg loading dose followed by 75 mg once daily for four weeks, at the discretion of the treating microsurgical team. The intended rationale was platelet inhibition in the early postoperative period; this regimen should not be interpreted as evidence of efficacy for venous congestion salvage.

Hourly Doppler and clinical flap evaluations were performed during the first 24 h by specialized staff in the postoperative care unit. By the following day, both vital signs and flap perfusion were satisfactory, allowing transfer to the surgical ward.

### Long-term outcome

On postoperative day 11, partial necrosis and breakdown of the intraoral skin paddle were identified (Figure 4A). The patient underwent revision surgery with removal of devitalized tissue and coverage using local intraoral tissue. The osseous component of the FFF remained viable.

**Figure 4.**
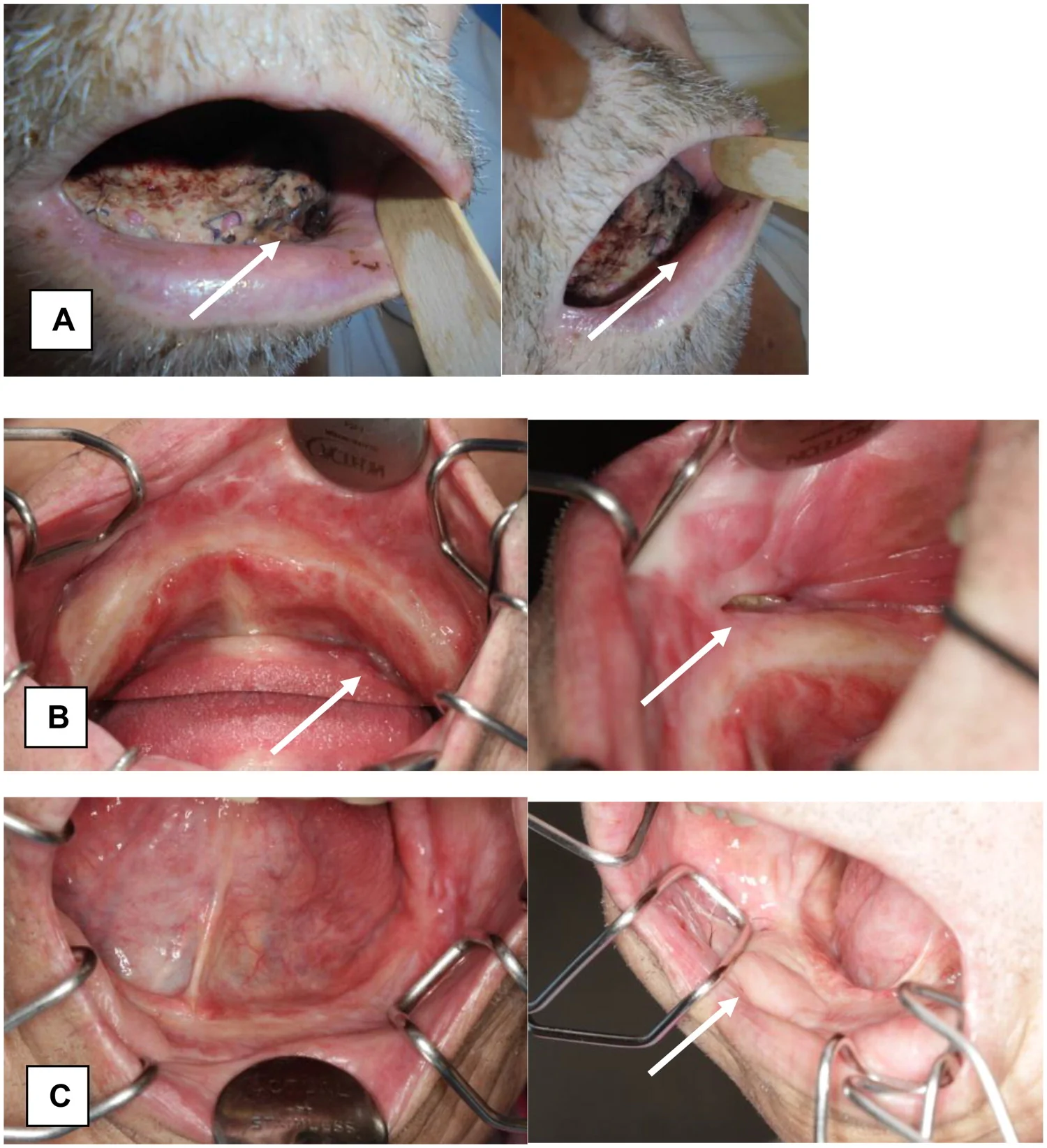
(A) Partial necrosis and breakdown of the de-epithelialized intraoral skin paddle on postoperative day 11 (white arrow). (B) Limited area of exposed neomandible with surrounding granulation tissue at follow-up (white arrow). (C) Healed intraoral soft tissue following secondary management, shown at the most recent follow-up (white arrow).

Following adjuvant radiotherapy, the patient was reviewed at 12 months after the index reconstruction. Intraoral healing was largely achieved, although a 0.5 cm area of exposed neomandible with surrounding granulation tissue was present (Figure 4B). Three months later, 1–2 cm of exposed neomandible was resected because of osteoradionecrosis. The remaining bone was viable and was subsequently covered with a nasolabial flap.

At the most recent follow-up, 6 months after secondary coverage with a nasolabial flap, the intraoral wound was healed and no further flap-related intervention had been required (Figure 4C). Dental implant rehabilitation was planned. Further follow-up was continued by the local oncology team; records from that follow-up were not available to the authors for the present report. Overall, the case resulted in survival of the osseous flap with clinically significant cutaneous-paddle necrosis and subsequent osteoradionecrosis requiring additional surgical management.

### Complication classification

The partial intraoral skin-paddle necrosis requiring operative debridement and local tissue coverage was classified as a Clavien–Dindo grade IIIb complication. The subsequent osteoradionecrosis requiring operative bone resection and nasolabial flap coverage was also classified as Clavien–Dindo grade IIIb. Functional outcomes were assessed clinically at follow-up. The patient was able to speak with mild difficulty. Oral intake was maintained, although he experienced weight loss that was not considered clinically severe. Mouth opening was moderately reduced. Standardized assessments of speech intelligibility, swallowing, mastication, oral intake, and mouth opening were performed and followed at the patient’s local hospital; however, these records were not available to the authors for the present report.

### Literature review

A scoping review was conducted following PRISMA-ScR guidelines to evaluate existing literature on salvage strategies for free fibular flaps (FFF) in patients with venous insufficiency. The review aimed to determine whether preoperative or postoperative guidelines exist for managing FFF in cases complicated by compromised venous networks in head and neck reconstruction.

### Search strategy

The search strategy was designed by a specialized health sciences librarian at Örebro University Library with expertise in systematic review methodology. Searches were conducted in Medline (Ovid), Embase (Elsevier), and the Cochrane Library (Wiley), with no restrictions on publication year.

### Eligible criteria

We included reports involving patients undergoing reconstruction with a free fibula flap in whom lower-limb varicose veins, chronic venous disease, peroneal venous varicosities, venous insufficiency, or other donor-site venous abnormalities potentially relevant to flap harvest or drainage were described.

We excluded studies involving congenital vascular malformations without reported acquired venous disease or a donor-site venous abnormality relevant to FFF harvest, reconstructions using flaps other than FFF, and reports focused exclusively on lower-limb arterial insufficiency.

All study designs were eligible if they included a defined patient population and reconstructive intervention. Non-English publications and conference abstracts were excluded because full clinical data could not be reliably assessed.

### Screening and data extraction

Abstract and full-text screening were performed in duplicate using Covidence (Covidence systematic review software, Veritas Health Innovation). MEDLINE was initially searched on 1 January 2026 and updated on 1 June 2026. Embase and the Cochrane Library were searched on 1 June 2026. The final screening dataset reflects the updated searches completed on 1 June 2026. Title, abstract screening and full-text assessment were performed independently by two reviewers, RT and S.H., using Covidence. Disagreements were resolved through discussion.

Data extraction was systematically conducted in Excel, capturing key variables: author, publication title, year, country of study, and study design (e.g. case report, case series, or retrospective cohort). The anatomical site of reconstruction was documented, with special reference to where the FFF was applied. Where available, reported salvage strategies, varicosities, and clinical outcomes were also recorded.

During screening, congenital vascular malformations were defined as developmental vascular anomalies described as venous, lymphatic, capillary, arteriovenous, or combined malformations. Acquired varicose veins and chronic venous disease were analysed separately and were not classified as congenital vascular malformations. Of the full-text articles assessed, 11 were excluded because they described congenital vascular malformations or involved the wrong patient population without relevant acquired donor-site venous disease.

## Results

The initial database search identified 168 studies. After removal of duplicates, 127 studies remained for title and abstract screening. Of these, 14 were selected for full-text review and assessed for eligibility. Ultimately, two studies met the inclusion criteria and were included in the final analysis (Table 1).

**Table 1.** Characteristics and reported management of the two publications included in the scoping review.

Study	Ng et al. 2014 [19]	Dziemianczyk-Pakiela et al. [20]
Journal	Journal of Reconstructive Microsurgery	Journal of Cranio-Maxillo-Facial Surgery
Country	Singapore	Poland
Study design	Letter reporting two cases	Retrospective cohort study
Site of reconstruction	Tibial reconstruction Mandibular reconstruction	Head-and-neck reconstruction
Surgical techniques	End-to-end anastomosis between one peroneal vein and the great saphenous vein One peroneal vein was ligated; the other was anastomosed end-to-side to the internal jugular vein.	All patients (*n* = 10) underwent surgery by the same surgeon. The peroneal artery was anastomosed to the superior thyroid artery or facial artery. One peroneal vein was anastomosed to a branch of the external or internal jugular vein using a single venous anastomosis.
Reported management and perioperative measures	The skin paddle and subcutaneous tissue were discarded, leaving a non-vascularized fibular bone graft wrapped in muscle. The fibula construct was dismantled and reshaped to a more convex configuration to reduce the risk of pedicle compression.	Perioperative thromboprophylaxis: subcutaneous enoxaparin sodium (Clexane) 0.4 mL, initiated 1 day before surgery and continued for 7 postoperative days; hydroxyethyl starch (HAES 10%), 500 mL daily for 7 postoperative days.
Venous findings	Varicose peroneal veins identified intraoperatively Varicose peroneal veins identified intraoperatively	Bilateral lower-limb superficial varicose veins were reported in the patient with flap failure.
Outcome	Venous thrombosis occurred; the skin paddle was discarded and the fibula was retained as a non-vascularized bone graft. Outcome not reported.	On postoperative day 5, venous thrombosis prompted revision of the anastomosis and intraoperative heparin flushing. Recurrent clinical signs of thrombosis occurred 2 days later and were confirmed by CTA. The flap was subsequently removed on postoperative day 7.
Comments	The authors suggested clinical examination and duplex ultrasonography when venous abnormalities are suspected. They discussed either selecting an alternative flap or proceeding with FFF reconstruction after individualized assessment. In the head-and-neck case, end-to-side anastomosis to the internal jugular vein was proposed as a technical option for donor-recipient vessel size and flow discrepancies. These observations were based on two cases and should be interpreted as descriptive rather than as a management recommendation.	In the flap-failure case, the peroneal artery-vein index was lower than in patients without complications. The authors proposed that marked artery-vein calibre discrepancy may be associated with venous thrombosis; however, this observation was based on a single flap failure and should be regarded as hypothesis-generating rather than as a validated predictive factor.

Two publications met the eligibility criteria. Together, they described three clinically relevant cases: two patients reported by Ng et al. [19] and one flap failure case within the cohort described by Dziemianczyk-Pakieła et al. [20]. Thus, ‘two studies’ refers to the number of publications, whereas ‘three cases’ refers to the number of individual cases relevant to the review question.

Ng et al. reported two cases in which varicose peroneal veins were identified intraoperatively during FFF harvest [19]. In the first case, venous thrombosis occurred in the setting of an unfavourable donor-recipient venous size relationship. The skin paddle and subcutaneous tissue were discarded, while the fibula was retained as a non-vascularized bone graft wrapped in muscle. In the second case, the fibula was reshaped to a more convex configuration to reduce the risk of pedicle compression; the reported outcome was not stated.

Dziemianczyk-Pakieła et al. reported a retrospective cohort of 10 patients undergoing head-and-neck reconstruction with FFF [20]. One patient, who had bilateral superficial varicose veins, developed donor-vein thrombosis and flap loss. That patient had a lower peroneal artery-vein index than the patients without complications. The report is hypothesis-generating only and does not establish the index as a predictive test or demonstrate that superficial varicose veins caused thrombosis.

In the flap-failure case, perioperative thromboprophylaxis consisted of subcutaneous enoxaparin sodium (Clexane) 0.4 mL initiated one day before surgery and continued for seven postoperative days, together with hydroxyethyl starch (HAES 10%) 500 mL daily for seven days. On postoperative day 5, venous thrombosis prompted revision surgery and intraoperative heparin flushing. Recurrent clinical signs of thrombosis developed two days later and were confirmed by CTA; the flap was removed on postoperative day 7. These reported perioperative pharmacological measures should not be interpreted as validated salvage strategies for chronic venous disease or established venous outflow obstruction [20].

## Discussion

This hybrid manuscript combines a surgical case report with a scoping review. The review identified only two publications comprising three relevant cases, and the evidence base was limited to small, heterogeneous reports. Accordingly, the available literature cannot establish a causal association between lower limb varicose veins and FFF venous thrombosis, flap compromise, or flap failure. Rather, the findings identify an important evidence gap regarding the relevance of donor-site venous assessment in selected patients.

In the study by Ng et al [19]. one patient developed venous thrombosis, while the surgical outcome of the second patient was not reported, leading to overall suboptimal results. In the study by Dziemianczyk-Pakiela et al [20], preoperative CTA was used to evaluate the peroneal artery-vein index in 10 patients. In the flap-failure case, the peroneal artery-vein index was lower than in patients without complications. This single observation raises a hypothesis regarding donor-recipient calibre discrepancy but does not establish an association with flap complications or failure.

The biological relationship between clinically visible superficial varicose veins and the deep peroneal veins used for flap drainage remains uncertain. Superficial varicose veins generally reflect acquired chronic venous disease involving the superficial venous system and do not, by themselves, demonstrate reflux, obstruction, thrombosis, valve dysfunction, or abnormal calibre in the deep peroneal veins. A plausible hypothesis is that abnormal donor-vein calibre, tortuosity, wall characteristics, compliance, valve function, or donor-recipient size mismatch could complicate venous anastomosis or promote flow disturbance in selected cases. However, these mechanisms were not directly measured in the present case and cannot be inferred from superficial varicosities alone. Dedicated venous duplex ultrasonography or other targeted venous imaging could be evaluated in future studies, but the current evidence is insufficient to recommend routine screening.

Regarding surgical techniques, the available data were limited. Ng et al [19]. reported using end-to-end anastomosis between a peroneal vein and the great saphenous vein in one case, and end-to-side anastomosis to the internal jugular vein in another, both sutured manually. In contrast, Dziemianczyk-Pakiela et al [20]. provided fewer technical details, noting only that the peroneal artery was anastomosed to the superior thyroid artery, and one peroneal vein was connected to branches of either the external or internal jugular vein *via* a single anastomosis.

Notably, both studies omitted critical details such as flap size and ischemia time, factors that are key indicators of flap viability and survival [21–24]. In the present case, the peroneal artery was anastomosed end-to-end to the facial artery, and both peroneal veins were connected to the facial vein and external jugular vein using microvascular couplers. Current evidence suggests that flap survival rates are comparable between sutured anastomoses and couplers, with the latter often reducing ischemia times [25–27].

The debate between single versus dual venous anastomosis remains unresolved. Some studies report equivalent survival rates for both approaches [28,29], while others advocate dual anastomoses for improved outcomes [30,31]. For example, Hanasono et al. observed increased blood velocity in single venous anastomoses, suggesting that improved drainage efficiency may negate the need for dual connections [32]. Conversely, Sulli et al. supports single venous anastomosis but recommends a dual approach in cases of venous congestion [33].

Nevertheless, the limited number of cases, both in the literature and in this report, is insufficient to draw firm conclusions about optimal surgical strategies for FFF in the setting of deep or superficial varicose veins. More robust clinical studies are needed to clarify best practices for flap selection, venous management, and salvage interventions in these challenging cases.

The diversity of surgical and pharmacological salvage options described in the literature highlights a substantial knowledge gap and the need for a deeper understanding of free flap physiology and survival. In the study by Ng et al. [19], streptokinase was not administered in one case because of the unsuitable diameter of the peroneal and recipient veins, as well as the presence of venous thrombosis within the FFF. Instead, the authors discarded the skin paddle and subcutaneous tissue, preserving the non-vascularized fibula bone graft wrapped in muscle. In their second case, they modified the fibula to increase its convexity, aiming to reduce the risk of pedicle compression.

In contrast, the second study reported a prophylactic pharmacologic strategy. Dziemianczyk-Pakiela et al. [20]. administered subcutaneous enoxaparin sodium (Clexane) 0.4 ml one day before surgery, followed by postoperative hydroxyethyl starch (HAES 10%) 500 ml daily for seven days.

In the present case, intraoperative venous congestion prompted revision of one venous anastomosis, heparinized saline irrigation, de-epithelialization of the skin paddle, and controlled drainage-hole creation. Subsequent clinical and ICG findings suggested improved perfusion, but the independent contribution of each measure cannot be determined. The osseous flap survived; however, partial skin-paddle necrosis required reoperation on postoperative day 11, and subsequent osteoradionecrosis required further surgical treatment. The case should therefore be interpreted as survival of the bony reconstruction with substantial soft-tissue and late radiation-associated complications, rather than as an uncomplicated or ‘excellent’ outcome.

Notably, neither of the two reviewed studies nor our case provides a structured or universally applicable salvage algorithm for compromised FFFs in the setting of varicosities. Aksoyler et al. proposed a surgical algorithm for managing vascular compromise in FFF based on 12 cases, but this sample remains too small for definitive recommendations [11]. The broad range of surgical and pharmacological salvage strategies reported, ranging from vein revisions and irrigation to pharmacologic anticoagulation, underscores the fragmented knowledge base and highlights the urgent need for evidence-based protocols.

From a surgical judgment perspective, caution is essential when pre-existing deep or superficial venous insufficiency or thrombosis is suspected. Yet, the literature offers limited guidance regarding both the reliability of FFF in such patients and clear strategies for complication management. Neither of the reviewed studies reported formal, replicable salvage protocols. There were no detailed intraoperative decision-making algorithms or structured postoperative measures such as pharmacological modulation, venous supercharging, or staged reconstruction specifically tailored to compromised venous anatomy. This absence of structured guidance represents a critical knowledge gap, especially considering that venous congestion remains one of the most frequent causes of flap failure in reconstructive microsurgery.

The need for guidance becomes even more pressing in oncology patients, who face a fourfold increased risk of thromboembolism, a risk that rises to 6.5 times higher when chemotherapy is involved [34]. Oncology patients may have an elevated risk of venous thromboembolism because of patient-, disease-, and treatment-related factors. However, the present case and the two identified publications [19,20], do not establish that superficial lower-limb varicose veins increase the risk of peroneal venous thrombosis or FFF failure. Any possible relationship between superficial venous disease, deep donor-vein characteristics, and flap outcome remains uncertain and requires dedicated investigation.

Currently, routine preoperative imaging tends to focus on arterial anatomy *via* CTA, MRA, or Doppler ultrasound, which may create a false sense of security if the venous system is not equally evaluated. In patients with marked clinical signs of chronic venous disease, previous venous interventions, previous venous thrombosis, or uncertain donor-site anatomy, individualized consideration of targeted venous assessment may be reasonable. However, the present case and the sparse literature do not support routine bilateral venous imaging, a specific imaging modality, or a standardized salvage protocol for all patients undergoing FFF reconstruction.

Interpretation of the postoperative course is also limited by important confounding factors. The patient had a 42-year smoking history, which may adversely affect wound healing and microvascular outcomes. In addition, adjuvant radiotherapy is a well-recognized contributor to impaired soft-tissue healing and osteoradionecrosis. These factors preclude attribution of skin-paddle necrosis or later bone exposure solely to pre-existing lower limb varicose veins or to an intraoperative venous abnormality.

## Limitations

This study has several limitations. Only two eligible publications were identified: a letter reporting two cases and a retrospective cohort study in which one clinically relevant flap-failure case was reported. This reflects the scarcity of published literature on salvage strategies for FFF in the presence of varicosities. This small evidence base restricts the external validity and generalizability of our findings.

The included studies reported heterogeneous outcome measures and varied in methodological rigor. Neither offered replicable salvage strategies or standardized outcome reporting, which limited the ability to synthesize findings meaningfully. Restricting the review to English-language studies may also have excluded potentially relevant data.

Additionally, there are inherent limitations to the scoping review methodology itself. By design, scoping reviews map the available literature rather than evaluate study quality, meaning that no formal risk of bias assessment or grading of evidence strength was performed.


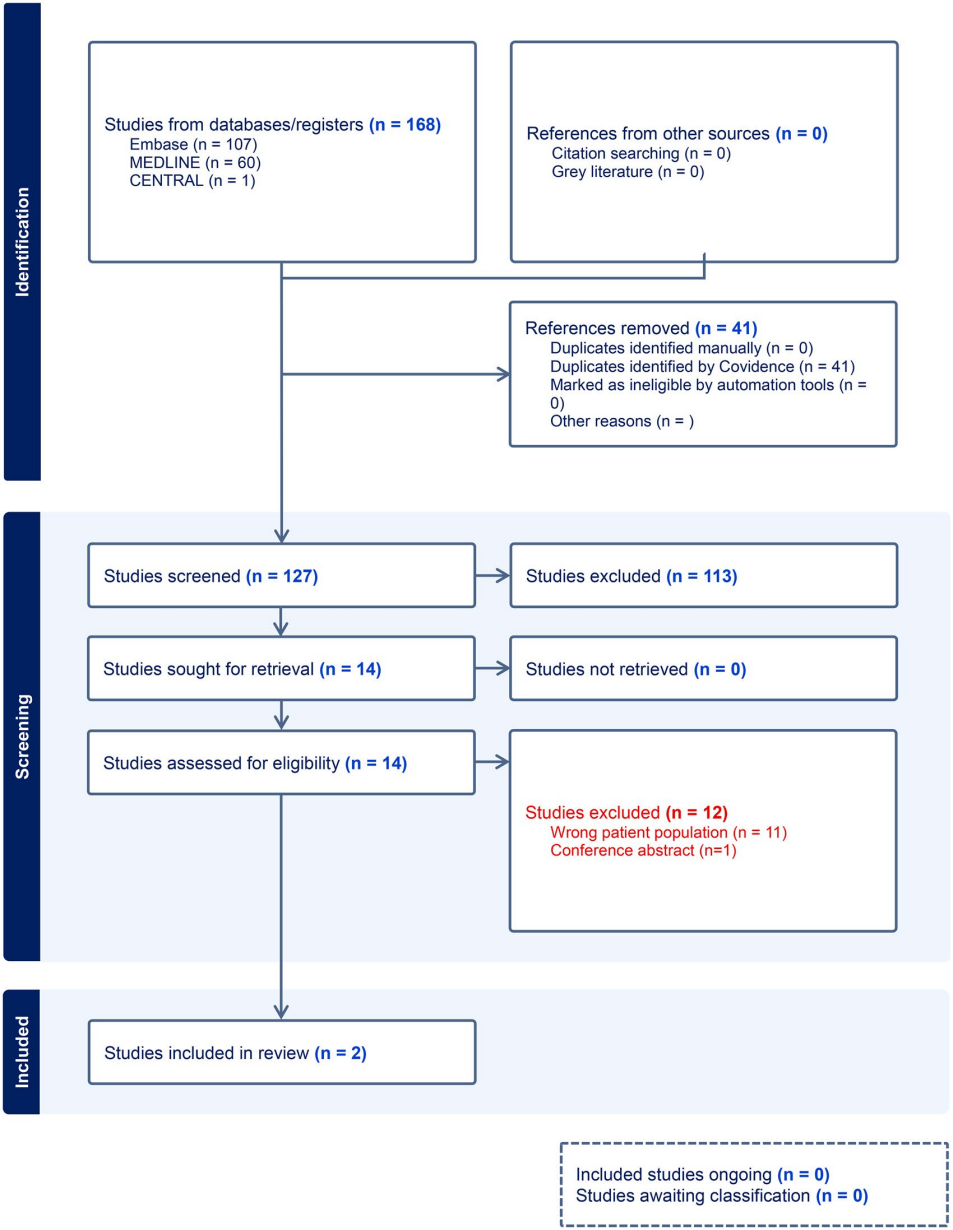


## Conclusion

Free fibula flap reconstruction in patients with lower-limb varicose veins or suspected donor-site venous abnormalities remains insufficiently characterized. In this case, intraoperative venous congestion was managed with anastomotic revision and local measures, resulting in survival of the osseous flap but partial skin-paddle necrosis and later osteoradionecrosis requiring additional surgery. The accompanying scoping review identified only two publications comprising three relevant cases and does not establish a causal relationship between superficial varicose veins and FFF failure.

Visible superficial varicosities should not be assumed to reflect disease of the deep peroneal venous system. Future prospective studies should define donor-site venous findings, assess the role of targeted preoperative venous imaging, and evaluate whether such assessment improves operative planning or clinical outcomes.

## Supplementary Material

Medline_revised.docx

## Data Availability

The data supporting the findings of this study are available from the corresponding author upon reasonable request. Access will be provided in accordance with the participant consent, and applicable data protection regulations.
