# Supplementary material for: Lower limb varicose veins in free fibula flap reconstruction: a surgical case report and scoping review
Source: Case Reports Plast Surg Hand Surg. 2026 Oct 7;13(1):2742738. doi: 10.1080/23320885.2026.2742738 (PMC13647389; doi:10.1080/23320885.2026.2742738)
Supplement: Supplementary file 1 [file ICRP_A_2742738_SM5593.docx]

**Database**(s): **Ovid MEDLINE(R) ALL** 1946 to June 01, 2026
**Host:** Ovid

(Initial search date: 2026-01-01, Updated search date: 2026-06-01, Coverage: 1946 to June 01, 2026)

**Date searched:** 2026-01-01

**Limits applied:** English language

| **Field Code** | **Definition** |
| --- | --- |
| / | Mesh-term |
| exp | exploded Mesh-term |
| ab | abstract |
| kf | keyword heading word |
| ti | Title |

| **Concept** | **#** | **Search** | **Results** |
| --- | --- | --- | --- |
| Fibula Flaps | 1 | Fibula/ or fibula*.ti,ab,kf. | 19936 |
|  | 2 | exp Surgical Flaps/ or ("flap*" or "graft*" or autograf* or transplant* or "surgically created structure*").ti,ab,kf. | 979069 |
|  | 3 | 1 and 2 | 6945 |
|  | 4 | Mandibular Reconstruction/ or ("mandib* reconstructi*" or "mandib* restoration*" or "maxillo-mandibular reconstructi*").ti,ab,kf. | 3316 |
|  | 5 | 3 or 4 | 8974 |
| Vascular malformations | 6 | exp Varicose Veins/ or Venous Insufficiency/ or Vascular Malformations/ or Arteriovenous Malformations/ or Arteriovenous Fistula/ or exp Phlebitis/ | 76716 |
|  | 7 | ("varicose vein*" or varicosities or "reticular vein*" or telangiectasias or "corona phlebectatica" or varix or varices or "chronic venous insufficienc*" or "venous stasis dermatitis" or lipodermatosclerosis or "venous ulcer*" or "venous hypertension" or "postphlebitic syndrome*" or "postthrombotic syndrome*").ab,kf,ti. | 41137 |
|  | 8 | ("venous reflux" or "valve incompetence" or "incompetent perforator vein*" or "saphenous vein insufficienc*" or "venous aneurysm*" or "vein aneurysm*" or "venous ectasia" or "vein ectasia" or "venous absence*" or "vein ectasia" or "venous hypoplasia" or "vein hypoplasia" or "venous dilatation*" or "vein ectasia" or "vein dilatation*" or phlebectasia or "venous malformation*" or "cavernous hemangioma*" or "venous dysplasia*" or "vein dysplasia*" or "venous anomal*" or "vein anomal*" or phlebangioma* or hemangioma* or "venolymphatic malformation*" or hemangioendothelioma* or phlebolith*).ab,kf,ti. | 36228 |
|  | 9 | ("vascular malformation*" or "klippel–trénaunay syndrome*" or "parkes weber syndrome*" or "blue rubber bleb nevus syndrome*" or "arteriovenous malformation*" or "arteriovenous fistula*" or "servelle–martorell syndrome*" or "vascular ehlers-danlos" or "may–thurner syndrome*" or "persistent sciatic vein*" or "anomalous popliteal vein*" or "malformation of calf vein*" or "venous duplication*" or "duplicated tibio-peroneal trunk*" or "duplicated popliteal vein*" or "popliteal vein entrapment syndrome*" or "cystic adventitial disease*" or "vascular anomal*" or "anomalous vein*" or phlebitis or vasculitis or venulitis).ab,kf,ti. | 99954 |
|  | 10 | 6 or 7 or 8 or 9 | 210644 |
| Concepts combined | 11 | 5 and 10 | 67 |
|  | 12 | limit 11 to English language | 60 |

**Database**: Embase.com

**Host:** Elsevier

**Date searched:** 2026-06-01

**Limits applied:** English

| **Field Code** | **Definition** |
| --- | --- |
| /de | Emtree term |
| /exp | exploded Entree term |
| ab, | abstract |
| kw | author keyword |
| ti, | title |
| /lim | limit |
| /it | Publication types |

| **Concept** | **#** | **Search** | **Results** |
| --- | --- | --- | --- |
| Fibula Flaps | 1 | 'fibula'/exp OR fibula*:ti,ab,kw | 24433 |
|  | 2 | 'surgical flaps'/exp OR 'flap*':ti,ab,kw OR 'graft*':ti,ab,kw OR autograf*:ti,ab,kw OR transplant*:ti,ab,kw OR 'surgically created structure*':ti,ab,kw | 1386112 |
|  | 3 | #1 AND #2 | 7893 |
|  | 4 | 'fibula graft'/de OR 'mandible reconstruction'/de OR 'fibula graft'/de OR 'mandible reconstruction'/de OR 'mandib* reconstructi*':ti,ab,kw OR 'mandib* restoration*':ti,ab,kw OR 'maxillo-mandibular reconstructi*':ti,ab,kw | 9413 |
|  | 5 | #3 OR #4 | 13399 |
| **Vascular malformations** | **6** | 'leg varicosis'/exp OR 'congenital blood vessel malformation'/de OR 'arteriovenous malformation'/de OR 'vein malformation'/de OR 'arteriovenous fistula'/de OR 'phlebitis'/de | 79781 |
|  | **7** | 'varicose vein*':ti,ab,kw OR varicosities:ti,ab,kw OR 'reticular vein*':ti,ab,kw OR telangiectasias:ti,ab,kw OR 'corona phlebectatica':ti,ab,kw OR varix:ti,ab,kw OR varices:ti,ab,kw OR 'chronic venous insufficienc*':ti,ab,kw OR 'venous stasis dermatitis':ti,ab,kw OR lipodermatosclerosis:ti,ab,kw OR 'venous ulcer*':ti,ab,kw OR 'venous hypertension':ti,ab,kw OR 'postphlebitic syndrome*':ti,ab,kw OR 'postthrombotic syndrome*':ti,ab,kw | 61287 |
|  | **8** | 'venous reflux':ti,ab,kw OR 'valve incompetence':ti,ab,kw OR 'incompetent perforator vein*':ti,ab,kw OR 'saphenous vein insufficienc*':ti,ab,kw OR 'venous aneurysm*':ti,ab,kw OR 'vein aneurysm*':ti,ab,kw OR 'venous ectasia':ti,ab,kw OR 'venous absence*':ti,ab,kw OR 'venous hypoplasia':ti,ab,kw OR 'vein hypoplasia':ti,ab,kw OR 'venous dilatation*':ti,ab,kw OR 'vein ectasia':ti,ab,kw OR 'vein dilatation*':ti,ab,kw OR phlebectasia:ti,ab,kw OR 'venous malformation*':ti,ab,kw OR 'cavernous hemangioma*':ti,ab,kw OR 'venous dysplasia*':ti,ab,kw OR 'vein dysplasia*':ti,ab,kw OR 'venous anomal*':ti,ab,kw OR 'vein anomal*':ti,ab,kw OR phlebangioma*:ti,ab,kw OR hemangioma*:ti,ab,kw OR 'venolymphatic malformation*':ti,ab,kw OR hemangioendothelioma*:ti,ab,kw OR phlebolith*:ti,ab,kw | 47243 |
|  | 9 | 'vascular malformation*':ti,ab,kw OR 'klippel–trénaunay syndrome*':ti,ab,kw OR 'parkes weber syndrome*':ti,ab,kw OR 'blue rubber bleb nevus syndrome*':ti,ab,kw OR 'arteriovenous malformation*':ti,ab,kw OR 'arteriovenous fistula*':ti,ab,kw OR 'servelle–martorell syndrome*':ti,ab,kw OR 'vascular ehlers-danlos':ti,ab,kw OR 'may–thurner syndrome*':ti,ab,kw OR 'persistent sciatic vein*':ti,ab,kw OR 'anomalous popliteal vein*':ti,ab,kw OR 'malformation of calf vein*':ti,ab,kw OR 'venous duplication*':ti,ab,kw OR 'duplicated tibio-peroneal trunk*':ti,ab,kw OR 'duplicated popliteal vein*':ti,ab,kw OR 'popliteal vein entrapment syndrome*':ti,ab,kw OR 'cystic adventitial disease*':ti,ab,kw OR 'vascular anomal*':ti,ab,kw OR 'anomalous vein*':ti,ab,kw OR phlebitis:ti,ab,kw OR vasculitis:ti,ab,kw OR venulitis:ti,ab,kw | 148367 |
|  | 10 | #6 OR #7 OR #8 OR #9 | 276864 |
| Concepts combined | 11 | #5 AND #10 | 119 |
|  | 12 | Limit English language | 107 |

**Database:** Cochrane Central Register of Controlled Trials

**Host:** Cochrane Library (Wiley)

**Date searched:** 2026-06-01

**Limits applied:**

| **Field Code** | **Definition** |
| --- | --- |
| ab | abstract |
| kw | keywords |
| ti | title |

| **Concept** | **#** | **Search** | **Results** |
| --- | --- | --- | --- |
| Fibula Flaps | 1 | (fibula*):ti,ab,kw | 802 |
|  | 2 | (flap* or graft* or autograf* or transplant* or "surgically created structure"):ti,ab,kw | 75388 |
|  | 3 | #1 and #2 | 158 |
|  | 4 | (mandib* NEXT reconstructi* or mandib* NEXT restoration* or maxillo-mandibular NEXT reconstructi*):ti,ab,kw | 117 |
|  | 5 | #3 or #4 | 235 |
| Vascular malformations | 6 | varicose NEXT vein* or varicosities or "reticular vein" or telangiectasias or "corona phlebectatica" or varix or varices or "chronic venous insufficiency" or "venous stasis dermatitis" or lipodermatosclerosis or "venous ulcer" or "venous hypertension" or "postphlebitic syndrome" or "postthrombotic syndrome" | 5546 |
|  | 7 | ("vascular malformation" or "klippel–trénaunay syndrome" or "parkes weber syndrome" or "blue rubber bleb nevus syndrome" or "arteriovenous malformation" or "arteriovenous fistula" or "servelle–martorell syndrome" or "vascular ehlers-danlos" or "may–thurner syndrome" or "persistent sciatic vein" or "anomalous popliteal vein" or "malformation of calf vein" or "venous duplication" or "duplicated tibio-peroneal trunk" or "duplicated popliteal vein" or "popliteal vein entrapment syndrome" or "cystic adventitial disease" or vascular NEXT anomal* or "anomalous vein" or phlebitis or vasculitis or venulitis):ti,ab,kw | 4755 |
|  | 8 | ("venous reflux" or "valve incompetence" or "incompetent perforator vein" or "saphenous vein insufficiency" or "venous aneurysm" or "vein aneurysm" or "venous ectasia" or "vein ectasia" or "venous absence" or "venous hypoplasia" or "vein hypoplasia" or "venous dilatation" or "vein dilatation" or phlebectasia or "venous malformation" or "cavernous hemangioma" or "venous dysplasia" or "vein dysplasia" or "venous anomaly" or "vein anomaly" or phlebangioma* or hemangioma* or "venolymphatic malformation" or hemangioendothelioma* or phlebolith*):ti,ab,kw | 946 |
|  | 9 | #6 or #7 or #8 | 10926 |
| Concepts combined | 10 | #5 and #9 | 1 |
